# Transcriptome Analysis of Acid-Responsive Genes and Pathways Involved in Polyamine Regulation in Iron Walnut

**DOI:** 10.3390/genes10080605

**Published:** 2019-08-10

**Authors:** Xiaomei Luo, Juncheng Liu

**Affiliations:** College of Forestry, Sichuan Agricultural University, Huimin Road 211 in Wenjiang District, Chengdu 611130, China

**Keywords:** RNA-seq, Short Time-series Expression Miner (STEM) analysis, acid soil stress, chlorophyll, photosynthesis

## Abstract

We reported changes in the co-regulated mRNA expression in iron walnut (*Juglans sigillata*) in response to soil pH treatments and identified mRNAs specific to acidic soil conditions. Phenotypic and physiological analyses revealed that iron walnut growth was greater for the pH 4–5 and pH 5–6 treatments than for the pH 3–4 and pH 6–7 treatments. A total of 2768 differentially expressed genes were detected and categorized into 12 clusters by Short Time-series Expression Miner (STEM). The 994 low-expression genes in cluster III and 255 high-expression genes in cluster X were classified as acid-responsive genes on the basis of the relationships between phenotype, physiology, and STEM clustering, and the two gene clusters were analyzed by a maximum likelihood (ML) evolutionary tree with the greatest log likelihood values. No prominent sub-clusters occurred in cluster III, but three occurred in cluster X. The Kyoto Encyclopedia of Genes and Genomes (KEGG) analysis indicated that acid-responsive genes were related primarily to arginine biosynthesis and the arginine/proline metabolism pathway, implying that polyamine accumulation may enhance iron walnut acid stress tolerance. Overall, our results revealed 1249 potentially acid-responsive genes in iron walnut, indicating that its response to acid stress involves different pathways and activated genes.

## 1. Introduction

*Juglans sigillata* Dode or iron walnut (whose nut shells have many pits and depressions in their surface) is an important species of walnut tree known for its production of edible nuts and hard wood. This species is sometimes grown in gardens and parks as an ornamental plant. It is commonly found in Yunnan but also in Guizhou, Sichuan, and Tibet in China and grows in soils that have a pH of approximately 4–6 [1]. Due to acid rain pollution and aluminium toxicity [2], the soils in the Long River region and in southern China have gradually become acidified. Acid soil stress affects tree growth and development and endangers the agronomic yields of trees, which have also evolved various molecular and physiological strategies to counteract acid soil stress. Therefore, an in-depth understanding of the gene expression regulatory mechanisms activated in response to acid stress is needed for efficient improvement.

Studies involving acid treatments have been conducted on various species across multiple kingdoms, including *Escherichia coli* [3], *Glycine max* [4], *Lactococcus lactis* [5], *Lactobacillus plantarum* [6], *Listeria monocytogenes* [7], and *Spinacia oleracea* [8]. The ability of plants to mitigate acid rain stress and sustain productivity may be related to the scavenging of stress-induced toxic oxygen species, such as hydrogen peroxide, hydroxyl radicals and superoxide radicals [9]. Catalases and peroxidases are two major enzyme groups whose members participate in the removal of hydrogen peroxide in plants [10,11]. Moreover, polyamines are well known for their anti-senescence and anti-stress effects because of their antioxidant properties as well as their acid-neutralizing, membrane-stabilizing, and cell-wall-stabilizing abilities [12,13,14,15,16,17]. Polyamines are maintained at optimal levels by antizymes and antizyme inhibitors and perform various cellular functions [18], but at high levels, these molecules are cytotoxic [19]. The stress response (including acid stress) as a potential mechanism may inhibit the proliferation of polyamine-depleted cells [20]. However, the molecular mechanism of how polyamines act in these processes is unclear, and plant acid tolerance remains elusive.

Little research is currently available on tree responses to acid stress. Studies involving iron walnut have focused mainly on the inhibitory effects of the extracts of its green husks [21], the chemical compositions and antioxidant capabilities of oils [22], speciation and phylogenetic relationships [23], its rapid Pleistocene diversification [24], the phenylpropanoid pathway [25], and anthocyanin biosynthesis [26]. Breeding for acid tolerance is particularly challenging because of the genetic complexity of this trait. Iron walnut has a complex genome (2n = 32) [27], ~606 Mb in size [28], and it is well documented that acid tolerance results from cooperative interactions among multiple morphological, physiological, and biochemical characteristics. In this study, we used transcriptome sequencing (transcriptome-seq) to identify differentially expressed genes (DEGs) in response to acid stress; accordingly, we used the leaf tissue of acid-resistant iron walnut landrace plants grown in different soil pH treatments. This analysis serves as a reference for future studies on iron walnut response to various stresses, such as drought, cold, and salt stresses.

## 2. Materials and Methods

### 2.1. Plant Growth under Acidic Soil Conditions

**Figure 1:**
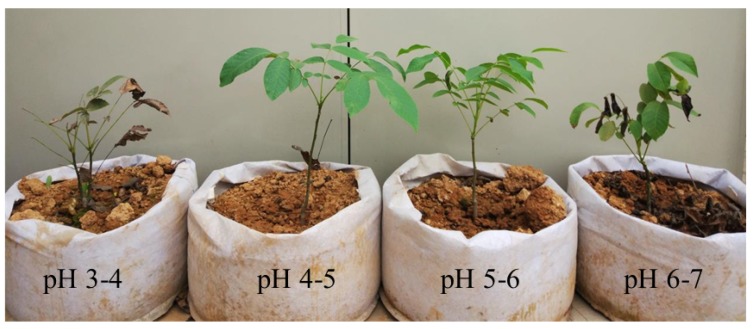
Iron walnut seedlings subjected to a series of soils of differing acidity.

An endemic iron walnut variety that is relatively acid tolerant was used for the analysis of acid tolerance-related genes. The pH of the original soil sample used for planting was measured and adjusted to generate a series of acidic soils (pH 3.25, pH 4.59, pH 5.26, pH 6.54) by adding the appropriate amount of calcium oxide (CaO). Twelve iron walnut seedlings were grown under the same conditions in a growth chamber (14 h photoperiod, 22 °C temperature, and 70% relative humidity; the soil pH was maintained at pH 3–4, pH 4–5, pH 5–6, or pH 6–7 during the acid stress treatment). One seedling was planted per pot, and three pots per soil pH treatment constituted one biological replicate. Seedlings approximately one year old were subjected to acid soil stress treatment for two months (Figure 1). Afterward, the leaf samples were first used to measure the net photosynthetic rate (Pn) on the basis of photosynthetically active radiation (PAR). The leaves were then harvested, flash frozen in liquid nitrogen and immediately stored at −80 °C for photosynthetic pigment and RNA sequencing (RNA-seq) analyses.

### 2.2. Contents of Photosynthetic Pigments and Pn-PAR Response Curves

To determine the effect of each pH treatment on photosynthesis, Pn-PAR response curves were generated in accordance with the non-rectangular hyperbolic photosynthetic model described by Farquhar et al. [29]. The chlorophyll and carotenoid contents were measured using the acetone-alcohol mixed extraction method described by Romero and Sanchez [30].

### 2.3. RNA Isolation and Iron Walnut RNA-Seq

RNA was extracted with an RNAprep Pure Plant Kit (Polysaccharides & Polyphenolics-rich) (Tiangen Biotech Co., Ltd., Beijing, China) according to the instructions provided. After extracting the total RNA and applying a DNase I treatment, we used magnetic beads with Oligo (dT) to isolate the mRNA. The mRNA molecules were fragmented at 150 bp, and cDNAs were synthesized via mRNA fragments as templates. The cDNA fragments were purified and resolved with EB buffer for end repair, single-nucleotide adenine (A) addition and adapter connections. After PCR amplification, an Agilent 2100 Bioanalyzer and an ABI Step One Plus Real-Time PCR System were used to quantify and qualify the sample library, respectively. The 150 bp library was then sequenced via an Illumina HiSeq™ 4000. Clean reads were obtained using Soap nuke software (http://www.seq500.com/ uploadfile/SOAPnuke.zip) by removing “dirty” reads containing adapter sequences, sequences with more than 10% unknown bases (“N”), and low-quality reads (reads with >50% of bases identified as low quality, where low-quality bases were defined as those whose sequencing quality was no more than 5). All of the clean reads were mapped against the genome data of walnut (ftp://ftp.ncbi.nlm.nih.gov/genomes/all/GCF/001/411/555/ GCF_001411555.1_wgs.5d) using HISAT2 software (version 2.1.0).

### 2.4. DEG Identification and Functional Annotation

Gene expression was calculated by the fragments per kilobase of exon model per million mapped reads (FPKM) method [31]. HTSeq software (version 0.10.0) was used to calculate the gene expression levels via a union model. The genes with FPKM values >1 were subjected to subsequent analysis. The DESeq method [32] was then used to identify DEGs based on a negative quadratic term distribution model. The raw counts and FPKM values were output by HTSeq, and the raw count of each gene was input into DESeq for differential expression analysis. The *q*-value of the DEGs was less than 0.05 (|Log_2_(FC)| ≥ 1, FC = fold change). R software (version 3.5.3) was used to construct and analyze Venn diagrams, as well as to analyze expression patterns and to perform a cluster analysis with the heatmap clustering method. The DEGs were ultimately annotated by the Gene Ontology (GO) and Kyoto Encyclopedia of Genes and Genomes (KEGG) databases via Omic Share tools (http://www.omicshare.com/).

### 2.5. Acid-Responsive Gene Identification and Enrichment Analysis

We compared the DEG profiles of plants under acid stress via STEM clustering [33]. Significant DEG profiles (*p* < 0.05) were further analyzed for acid-responsive genes based on the relationship between these gene expression profiles and those of iron walnut seedlings subjected to different soil pH treatments. Molecular phylogenetic analysis of these acid-response-related genes was also performed with MEGA7 [34]; the sequences were aligned by ClustalW, and molecular phylogenetic relationships were inferred by the maximum likelihood (ML) method based on the Tamura–Nei model [35]. Initial trees for the heuristic search were obtained automatically by applying the neighbor-joining and Bio NJ algorithms to a matrix of pairwise distances that were estimated using the maximum composite likelihood approach and then selecting the topology with the superior log likelihood value. The tree is drawn to scale, and the branch lengths represent the number of substitutions per site. The acid-responsive genes related to different biological functions were classified and grouped via GO analysis. KEGG pathway analysis was also used to identify biological pathways of the acid-responsive genes.

### 2.6. Quantitative Real-Time PCR (qRT-PCR) Validation

To validate the results of the RNA-seq data, qRT-PCR was performed to analyze the expression of genes with SYBR Green I using the iron walnut 18S gene as a standard control [36]. cDNAs were reverse-transcribed using a True Script First Stand cDNA Synthesis Kit (Aidlab, Beijing, China). Reverse transcription was performed in a total volume of 20 μL, which consisted of 1 μL of RNA, 4 μL of 5× RT Reaction Mix, 0.8 μL of Rondam primer/oligo (dT), 0.8 μL of True Script H^-^RTase/RI Mix, and 13.4 μL of RNase-free dH_2_O. The reaction tubes were maintained at 42 °C for 40 min and then at 65 °C for 10 min. The cDNAs were then used as templates for real-time PCR using gene-specific primers. Real-time PCR was conducted in a total volume of 10 μL, which consisted of 5 μL of 2× SYBR^®^ Green Supermix, 1 μL of primers, 1 μL of cDNA, and 3 μL of ddH_2_O. The reaction conditions were 95 °C for 3 min, followed by 39 cycles of 95 °C for 10 s and 57–61 °C for 30 s. The relative quantification of gene expression was determined by qRT-PCR software 3.0 using the Pfaffl method. Mean values and standard errors were calculated from three independent experiments consisting of three biological replicates of iron walnut, and the data were normalized to the relative efficiency of each primer pair.

## 3. Results

### 3.1. Determination of Pn-PAR Response Curves and Photosynthetic Pigment Contents

**Figure 2:**
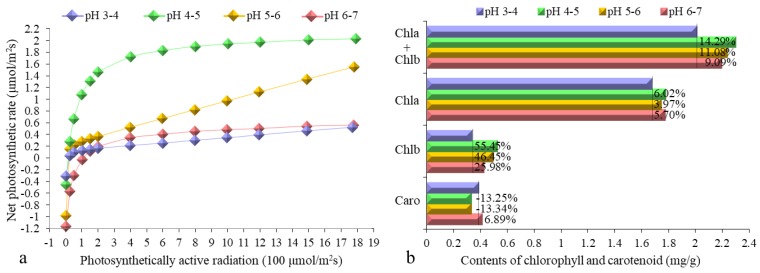
Pn-PAR response curves (**a**) and contents of chlorophyll and carotenoids (**b**) of *J. sigillata* seedlings subjected to soils with different pH values. The percentage in the top bar in (**b**) shows the percentage increase for each treatment compared with the pH 3–4 treatment. The four colors indicate different pH values: pH 3–4 (purple), pH 4–5 (green), pH 5–6 (orange), and pH 6–7 (red). Chla—chlorophyll a, Chlb—chlorophyll b, Caro—carotenoid, Pn—net photosynthetic rate, PAR—photosynthetically active radiation.

To investigate the effects of each pH iron walnut in such pH treatment, Pn-PAR response curves were generated (Figure 2a). The trend of the Pn-PAR response curves for the iron walnut seedlings was as follows: pH 4–5 > pH 5–6 > pH 6–7 > pH 3–4. The photosynthetic pigment contents were also measured (Figure 2b). The total contents of chlorophyll a and b were 14.29%, 11.08%, and 9.09% greater for the iron walnut seedlings at pH 4–5, pH 5–6, and pH 6–7 than at pH 3–4, respectively. Similarly, the content of chlorophyll b was 55.45%, 46.45%, and 25.98% greater for the iron walnut seedlings at pH 4–5, pH 5–6, and pH 6–7, respectively, than at pH 3–4. Moreover, the content of chlorophyll a was 6.02%, 3.97%, and 5.70% greater for the iron walnut seedlings at pH 4–5, pH 5–6, and pH 6–7 than at pH 3–4, respectively. However, the carotenoid content showed the opposite trend from the total chlorophyll content; it was 13.25% and 13.34% lower for the iron walnut seedlings at pH 4–5 and pH 5–6, respectively, but 6.89% greater in the iron walnut seedlings at pH 6–7. In total, the physiological data showed that the growth of iron walnut was greater in the pH 4–5 and pH 5–6 treatments than in the pH 3–4 and pH 6–7 treatments.

### 3.2. Illumina Sequencing of Different cDNA Libraries

cDNA libraries were constructed from leaves harvested two months after acid stress; three biological replicates were sequenced using the Illumina HiSeq™ 4000 platform. After cleaning and checking the read quality, we obtained nearly 32.87 million 150 bp paired-end clean reads. Among them, all of the Q30 (Phred score) values were greater than 94%, and the GC content was greater than 45% in each sample. The statistical data of the sequenced samples are shown in Table 1. The clean reads of each sample were mapped to the full gene set of walnut. The transcriptome data from 12 samples mapped to 27,316 genes, which covered 74.11% of the whole gene set. The major mapping reads indicated reliable transcriptome data.

### 3.3. DEG Analysis and Validation of the Iron Walnut Sequencing Data

**Figure 3:**
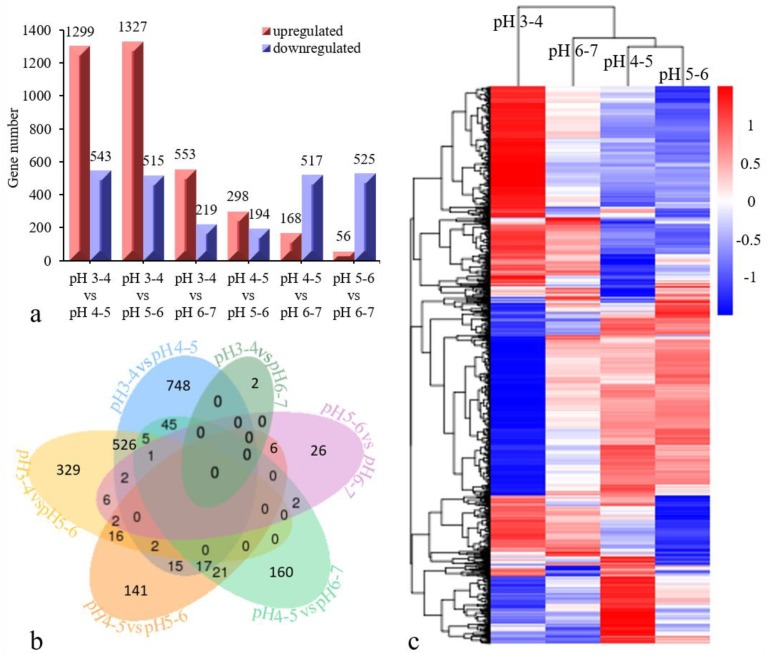
Comparison of differentially expressed genes (DEGs) in seedlings subjected to different acid stresses. (**a**) The *x*-axis represents each comparison treatment, and the *y*-axis presents the gene number. The red color indicates upregulated genes, and the purple color indicates downregulated genes. (**b**) A Venn diagram shows the overlap between DEGs that are responsive to acid stress treatments. (**c**) the gene expression patterns and cluster analysis results of twelve samples are shown. The red/blue color on the far right indicates the up/down regulated DEGs of each sample.

**Figure 4:**
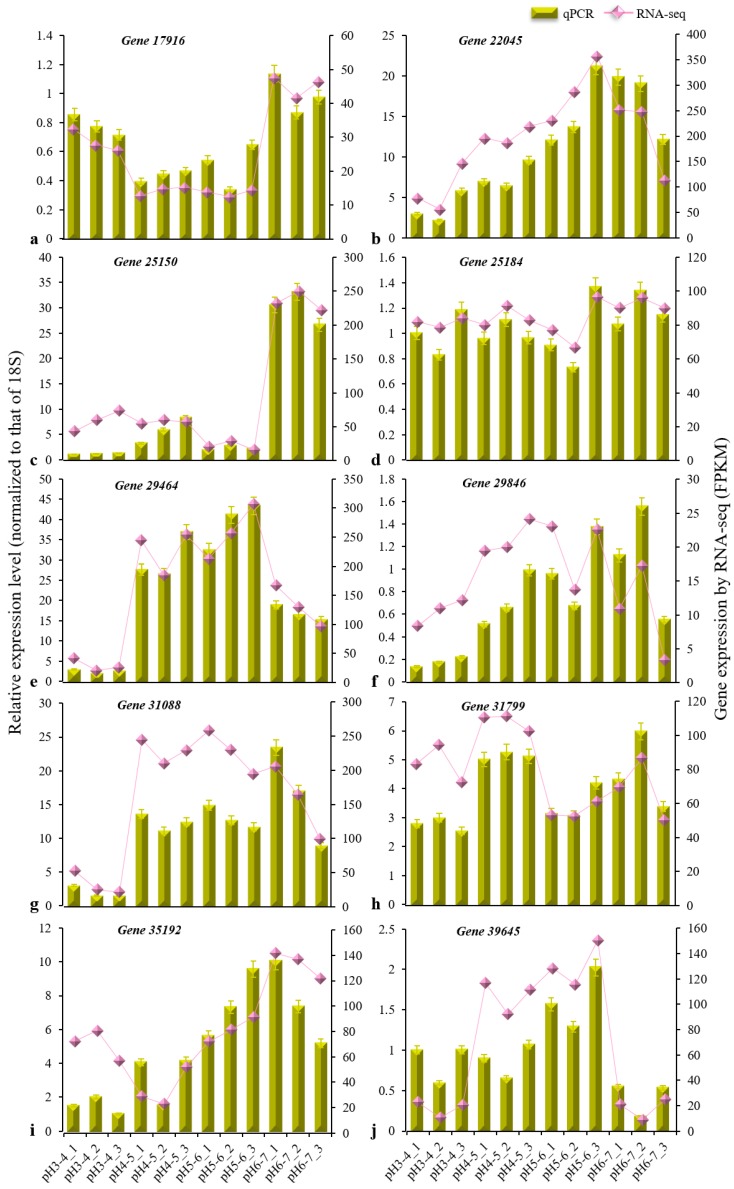
Expression of 10 DEGs (**a**–**j**) in response to acid stress treatments. The vertical bar charts with simple error bars (left *y*-axis) represent the quantitation of 10 gene transcripts among twelve samples via qRT-PCR. The values are the means ± SDs (*n* = 3). The line and scatter plot (right *y*-axis) represent the transcript abundance (FPKM) of twelve samples of each gene, as revealed by RNA-seq. The three replicate samples per treatment were treated with soils of pH 3–4, 4–5, 5–6, and 6–7.

The gene expression in each sample is presented in Table S1. After removing redundant DEGs between six comparison groups, a total of 2768 DEGs (q < 0.05) were identified based on a pairwise comparison analysis (Figure 3a). Six cross-comparisons between the different gene sets were illustrated in a Venn diagram (Figure 3b). A cross-comparison of pH 3–4 vs. pH 4–5 showed that the most-expressed genes (748) were unique to the other cross-comparisons, while pH 3–4 vs. pH 6–7 resulted in fewer uniquely expressed genes (2). The heatmap clustering showed the gene expression patterns for the 2768 DEGs to be clustered into three categories (Figure 3c). The DEGs in the pH 3–4 treatment differed from those in the other three treatments (pH 4–5, pH 5–6, pH 6–7), and the DEGs in the pH 6–7 treatment also differed from those in the other two treatments (pH 4–5, pH 5–6). Among these DEGs, ten were confirmed via qRT-PCR to be distinctly differentially expressed between treatments (Table 2, Figure 4). The results of this experiment were essentially consistent with the RNA-seq data.

### 3.4. GO-and KEGG-Annotated DEGs

**Figure 5:**
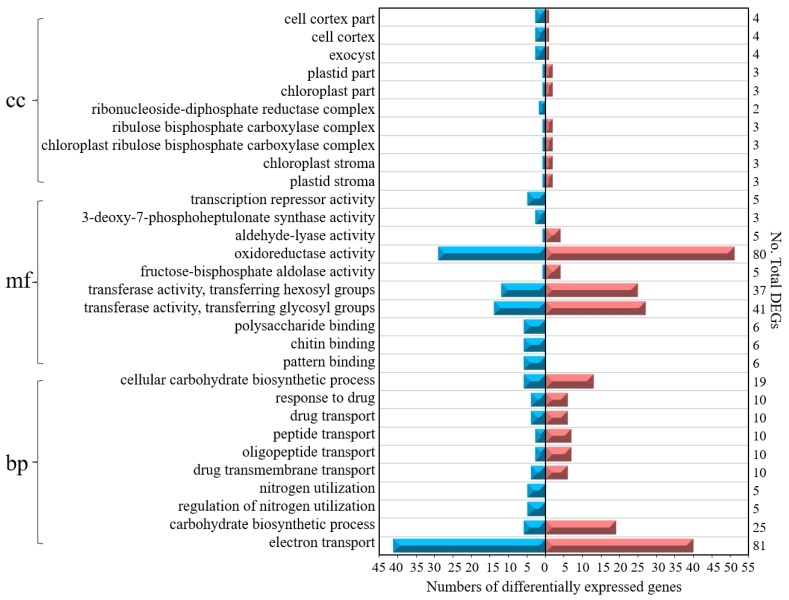
Top ten Gene Ontology (GO) term classifications of the DEGs from six cross-comparisons between the different gene sets. BP—biological process; MF—molecular function; CC—cellular component. The blue color on the left indicates downregulated DEGs, while the red color on the right indicates upregulated DEGs.

The top ten GO term classifications of the DEGs from six cross-comparisons between the different gene sets are illustrated in Figure 5. Among these GO terms, 45% of DEGs belong to the biological process category, 47% of DEGs belong to the molecular function category, and 8% of DEGs belong to the cellular component category. In fact, 40% of the DEGs were associated with electron transport (81 DEGs) and oxidoreductase activity (80 DEGs), 6% of DEGs were associated with 3-deoxy-7-phosphoheptulonate synthase activity (3 DEGs), plastid stroma (3 DEGs), chloroplast stroma (3 DEGs), the chloroplast ribulose bisphosphate carboxylase complex (3 DEGs), the ribulose bisphosphate carboxylase complex (3 DEGs), the ribonucleoside-diphosphate reductase complex (2 DEGs), chloroplast parts (3 DEGs), and plastid parts (3 DEGs).

Thirty-eight KEGG pathways (false discovery rate (FDR) < 0.05) from six cross-comparisons between the different gene sets are listed in Table 3. Approximately 85% of the DEGs were assigned to metabolism, and the remaining 15% of the DEGs were nearly equally assigned to genetic information processing, environmental information processing, and organismal systems. In fact, 25% of the DEGs focused on metabolic pathways (229 DEGs), and 1% of the DEGs were associated with the proteasome (1 DEG), other glycan degradation (1 DEG), the mRNA surveillance pathway (1 DEG), the biosynthesis of unsaturated fatty acids (1 DEG), and isoflavonoid biosynthesis (1 DEG).

### 3.5. STEM Cluster Analysis of DEGs and Identification of Acid Resistance-Related Genes

**Figure 6:**
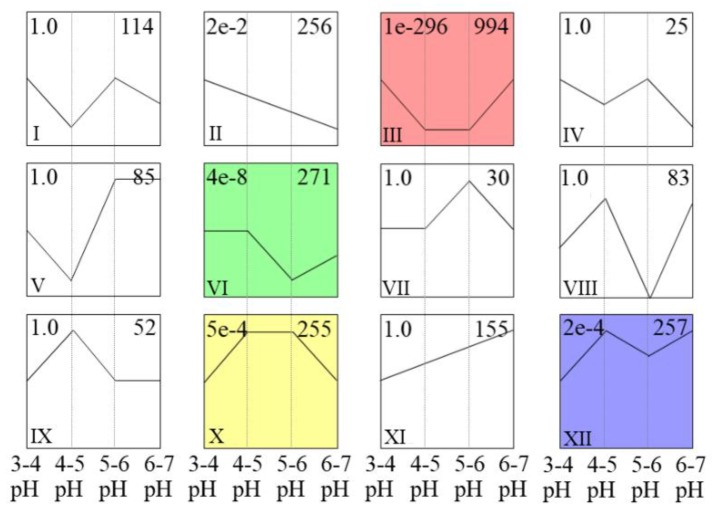
Comparison of DEG profiles in response to acid stress as determined by STEM clustering. For each profile, the top-left number indicates the *p*-value, the bottom-left number indicates the profile ID, and the top-right number indicates the genes assigned to this profile. The color profile indicates *p* < 0.05.

STEM cluster analysis was used to estimate all of the DEGs, as shown in Figure 6 and Table S2. A total of 2768 DEGs were filtered, and 2577 DEGs ultimately formed 12 clusters. Four clusters were significantly related to pH treatment (clusters III, VI, X, XII). Furthermore, all 994 genes in cluster III were clearly expressed at low levels in the pH 4–5 and pH 5–6 treatments, while all 255 genes in cluster X were clearly expressed at high levels in the pH 4–5 and pH 5–6 treatments. In these experiments, iron walnut plants grew better in the pH 4–5 and pH 5–6 treatments than in the pH 3–4 and pH 6–7 treatments. Moreover, the physiological data showed that the growth of iron walnut was greater in the pH 4–5 and pH 5–6 treatments than in the pH 3–4 and pH 6–7 treatments. Hence, the 1249 genes in cluster III and cluster X were found to be potential acid-responsive genes.

### 3.6. Molecular Phylogenetic Analysis of Acid Resistance-Related Genes

**Figure 7:**
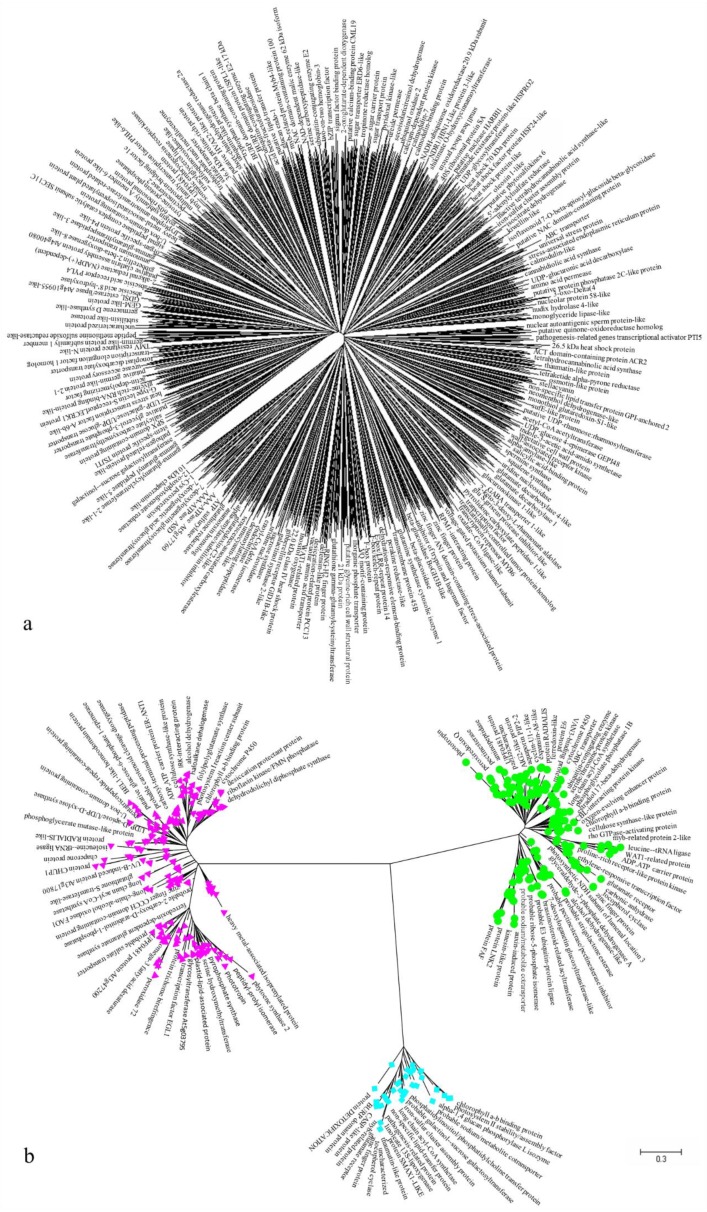
Evolutionary analyses of cluster III (**a**) and cluster X (**b**) conducted with MEGA7. The evolutionary history was inferred using the maximum likelihood (ML) method based on the Tamura–Nei model. Initial tree(s) for the heuristic search were obtained automatically by applying the neighbor-joining and Bio NJ algorithms to a matrix of pairwise distances that were estimated using the maximum composite likelihood approach and then selecting the topology with the superior log likelihood value. The tree is drawn to scale, and the branch lengths represent the number of substitutions per site. All positions containing gaps and missing data were eliminated. The red color represents the cluster including 194 sequences, while the green color represents another cluster including 193 sequences, the blue color represents the rest cluster including 36 sequences.

Evolutionary analyses of the 994 DEGs in cluster III and the 255 DEGs in cluster X were conducted with MEGA7 (Figure 7). The trees with the highest log likelihood values are shown in Figure 7a,b (−2,370,992.00 and −120,896.32). The 994 low-expression DEGs in cluster III included 1339 nucleotide sequences, as shown in Figure 7a, and the 255 high-expression DEGs in cluster X included 423 nucleotide sequences, as shown in Figure 7b. The total length of the combined sequences in cluster III is 7840 bp and that in cluster X is 5416 bp after alignment by ClustalW in Mega 7. All positions with gaps and missing data were eliminated, then we obtained 1017 bp in cluster III, and 1964 bp in cluster X. There was a total of 208 positions (Figure 7a) and 258 positions (Figure 7b) in the final dataset. No prominent sub-clusters were observed in cluster III (Figure 7a), but three notable sub-clusters were observed in cluster X (Figure 7b). Two long nucleotide sequences with nearly the same number of nucleotides were observed in the green (193 sequences) and red (194 sequences) sub-clusters. Thirty-six nucleotide sequences formed the small blue sub-cluster. Interestingly, each sub-cluster in cluster X contained sequences related to photosynthesis and chlorophyll (Figure 7b). In total, differences occurred within high-expression DEGs in cluster X but not within low-expression DEGs in cluster III.

### 3.7. Gene Function and Pathway Enrichment Analyses of Acid Resistance-Related Genes

**Figure 8:**
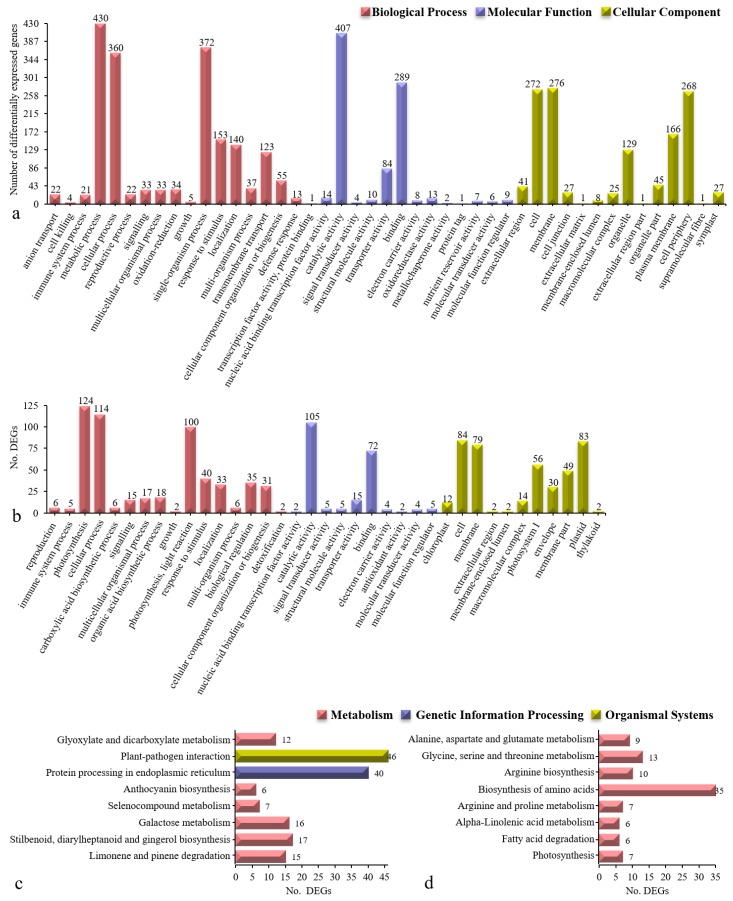
GO terms and Kyoto Encyclopedia of Genes and Genomes (KEGG) pathway distributions in cluster III (**a**,**c**) and cluster X (**b**,**d**). In (**a**,**b**)**,** *x*-axis indicates each GO term, and the *y*-axis indicates the number of DEGs in (**c**) and (**d**) indicates the number of DEGs, and the *y*-axis indicates each pathway. The three colors in (**a**,**b**) indicate the GO terms belonging to the biological process (red), molecular function (purple), and cellular component (light green) categories. The three colors in the pathways in (**c**) and (**d**) represent metabolism (red), genetic information processing (purple), and organismal systems (light green). The number above/to the right of each bar indicates the number of DEGs assigned to that GO term or pathway.

The combination of the above strategies resulted in final totals of 994 and 255 DEGs. We then performed GO and KEGG enrichment analyses of these two groups of genes (Figure 8). The assigned GO functions of the genes in cluster III and cluster X broadly encompassed 45 and 37 functional categories, respectively. The 994 low-expression DEGs in cluster III prominently included the GO terms cell periphery, plasma membrane, oxidoreductase activity, transporter activity, anion transport, oxidation-reduction process, transmembrane transport, and defence response (Figure 8a). The 255 high-expression DEGs in cluster X were represented by the GO terms thylakoid, chloroplast, plastid, photosystem I, envelope, extracellular region, photosynthesis, light reaction, organic acid biosynthetic process, and carboxylic acid biosynthetic process (Figure 8b). Approximately half of the GO functional enrichment terms overlapped between clusters III and X, including response to stimulus, binding, and extracellular region. Furthermore, oligopeptide transport, peptide transport, chitin binding, oxidoreductase activity, chloroplast stroma, and cell cortex were involved in the scavenging of stress-induced toxic oxygen species and polyamine regulation.

The KEGG pathway analysis revealed that the 994 low-expression DEGs in cluster III were involved mainly in eight pathways: Limonene/pinene degradation, stilbenoid/diarylheptanoid/ gingerol biosynthesis, galactose metabolism, selenocompound metabolism, anthocyanin biosynthesis, glyoxylate/dicarboxylate metabolism, protein processing in the endoplasmic reticulum, and plant-pathogen interactions (Figure 8c). The analysis also revealed that the 255 high-expression DEGs in cluster X were involved mainly in another eight pathways: Photosynthesis, fatty acid degradation, α-linolenic acid metabolism, biosynthesis of amino acids, alanine/aspartate/glutamate metabolism, glycine/serine/threonine metabolism, arginine biosynthesis, and arginine/proline metabolism (Figure 8d). There were no overlapping KEGG pathways between clusters III and X. Among these pathways, glyoxylate/dicarboxylate metabolism, biosynthesis of amino acids, alanine/aspartate/glutamate metabolism, glycine/serine/threonine metabolism, arginine biosynthesis, and arginine/proline metabolism were involved in polyamine metabolism and regulation.

Six genes closely related to polyamine biosynthesis/metabolism (*gene42780*, *gene28192*, *gene4089*, *gene31260*, *gene24987*, *gene9388*) are listed in Table 4. They are described as spermidine hydroxycinnamoyl transferase-like, spermine synthase-like, S-adenosylmethionine synthase 1-like, arginine decarboxylase-like, serine/arginine-rich splicing factor RS40-like, and mitochondrial arginine transporter BAC2-like, which were associated with six GO terms (molecular function, catalytic activity, organic acid biosynthetic process, carboxylic acid biosynthetic process, binding, transport), and 12 KEGG pathways (biosynthesis of secondary metabolites; metabolic pathways; stilbenoid, diarylheptanoid and gingerol biosynthesis; flavonoid biosynthesis; phenylpropanoid biosynthesis; β-alanine metabolism; glutathione metabolism; cysteine and methionine metabolism; arginine and proline metabolism; biosynthesis of amino acids; spliceosome; thermogenesis).

## 4. Discussion

Acidic soil is prevalent in the Yangtze valley and southern area of China. Among the major trees of economic value, iron walnut is relatively tolerant to acid stress and therefore is often grown on more marginal sites. As a landrace, iron walnut has developed a strong tolerance and adaptability to acid stress. Using high-throughput RNA-seq technology, we compared in detail the transcriptional differences and overlap in response to different levels of soil pH and identified the changes in gene expression in the iron walnut seedlings during the whole acid response process.

### 4.1. Acid Resistance-Related Gene Identification

In this study, the mRNA of iron walnut seedlings exhibiting good acid tolerance was sequenced using an Illumina HiSeq™ 4000 platform with Sera-Mag Magnetic Oligo-dT Beads. A clear bioinformatic map of the mRNAs involved in multiple biological processes was produced. As a result, 32.87 million clean reads were collected from 12 samples of plants subjected to different soil pH treatments; this number met the requirements for further analysis. The clean Q30 reads constituted more than 94% of the total, suggesting high-quality sequencing. HISAT2 software was used to BLAST the transcriptome data against the walnut genome; 74% of the reads were mapped to the reference genome. The major mapping reads indicated reliable transcriptome data. These non-mapped tags most likely represent regions where the reference genome is incomplete [37]. Alternatively, there are genetic differences between the reference genome and the iron walnut seedlings used in this study [28]. Moreover, the RNA-seq data for reference genome annotations might not represent all major tissue types, developmental stages, or statuses during responses to abiotic and biotic stresses [38].

The global analysis of the DEGs provided a comprehensive dataset on the response to acid soil stress in the leaves of iron walnut seedlings. We identified 12 clusters for all DEGs and obtained four clusters that were significantly related to soil pH (*p* < 0.05). The genes in cluster III and cluster X were determined to be potential acid-responsive genes on the basis of the relationship between these gene expression profiles (STEM clustering trends) and the growth of the iron walnut seedlings subjected to the pH 4–5 and pH 5–6 treatments (the phenotype), as well as the Pn-PAR response curves and photosynthetic pigment contents (physiological data). Similarly, potential genes in both maize and humans have also been identified by gene expression profiling and phenotyping [39,40,41].

### 4.2. Analysis of Acid Resistance-Related Genes

The acid resistance-related genes in clusters III and X were further analyzed by the ML method. No prominent sub-clusters were observed in cluster III, but three notable sub-clusters were observed in cluster X, indicating a large difference between the low-expression genes in cluster III and the high-expression genes in cluster X. Two nearly identical large sub-clusters and a small sub-cluster in cluster X all contained sequences related to photosynthesis and chlorophyll, indicating that differences in nucleotide sequences occurred within the high-expression genes in cluster X, although these nucleotide sequences have similar biological functions. A similar phenomenon was also found in *Arabidopsis thaliana* [42], *Capsicum annuum* [43], *Lactobacillus kefiranofaciens* [44], *Rana arvalis* [45], and *Zea mays* [46].

The GO functional terms, including plasma membrane, oxidoreductase activity, anion transport, oxidation-reduction process, transmembrane transport, and defence response, were the most affected among the terms associated with the low-expression genes in cluster III, indicating that the genes in these different functional categories were predicted to be involved at low expression levels in iron walnut seedlings. GO terms including chloroplast, photosystem I, photosynthesis, and light reaction were associated with the most strongly affected, high enough genes in cluster X, indicating that the genes associated with these different functional categories can be predicted to be involved at high expression levels in iron walnut seedlings. GO terms such as photosystem I and photosynthesis were reported by Shikanai et al. [47], Guan et al. [48], and Otani et al. [49]. Approximately half of the GO functional enrichment terms overlapped between clusters III and X, including response to stimulus, binding, and extracellular region, suggesting that these associated genes can be predicted to be involved in essential plant biological processes [17,50,51]. Several similar GO terms, such as oligopeptide transport, peptide transport, chitin binding, oxidoreductase activity, chloroplast stroma, and cell cortex, have been identified as being associated with anti-senescence and anti-acid stress effects in *A. thaliana*, *C. annuum*, *Nicotiana tabacum*, *Triticum aestivum*, and *Z. mays* [11,12,16,40,46,52], implying that these GO terms are involved in the scavenging of stress-induced toxic oxygen species and polyamine regulation.

KEGG pathway analysis in this study revealed sixteen pathways that were significantly affected by acid soil stress. The low-expression genes associated with eight pathways, glyoxylate/ dicarboxylate metabolism, limonene/pinene degradation, plant-pathogen interaction, etc., in cluster III significantly differed throughout the whole acid soil stress period, implying that these genes are involved in functions of defence, antioxidation, free radical scavenging, sugar, lipids as an energy source, protein processing, and polyamine metabolism and regulation. The expression of high-expression genes associated with another eight pathways (alanine/aspartate/glutamate metabolism, arginine biosynthesis, arginine/ proline metabolism, etc.) in cluster X significantly varied throughout the whole acid soil stress period. These results indicated that polyamine accumulation enhanced iron walnut acid stress tolerance. Similar studies by Gupta et al. [11] reported that the accumulation of polyamines occurs under many types of abiotic stress, including acid stress, and modulation of their biosynthetic pathway results in tolerance to stress. Hussain et al. [9] also reported a strong correlation between the presence of polyamines and improved tolerance to environmental stresses (acid drought, salinity, low temperature, oxidative stress and metal toxicity). Taie et al. [16] reported that polyamines promote growth and antioxidant activity and protect genomic DNA in heavy metal-stressed wheat plants. Similarly, Liu et al. [53] noted that polyamines provided environmental stress tolerance to plants. Majumdar et al. [46] demonstrated that polyamines contributed to the resistance of maize against *Aspergillus flavus* infection and to aflatoxin production. Amrani et al. [15] revealed that polyamines improved sucrose-induced tolerance to atrazine-mediated chemical stress in *A. thaliana*. Furthermore, Velikova et al. [4] proposed that exogenous polyamines could be attributed to protecting acid rain-treated bean plants via acid-neutralizing and antioxidant effects, as well as by their ability to stabilize membranes by associating with negatively charged phospholipids.

### 4.3. Hypothetical Regulatory Mechanisms of Acid Resistance

We further analyzed six genes related to arginine biosynthesis and the metabolic pathways of acid stress responses, which included genes related to arginine, ornithine, agmatine, methionine, putrescine, spermidine, and spermine. *Gene42780*, “spermidine hydroxycinnamoyl transferase-like”, plays a role in producing tricoumaroyl-, tricaffeoyl-, and triferuloyl-spermidine [54]. *Gene28192*, “spermine synthase-like”, plays a role in producing spermine from spermidine [11]. *Gene4089*, “S-adenosylmethionine synthase 1-like”, plays a role in producing spermine and spermidine from putrescine [9]. *Gene31260*, “arginine decarboxylase-like”, plays a role in producing agmatine from L-arginine [55,56]. *Gene24987*, “serine/arginine-rich splicing factor RS40-like”, and *Gene9388*, “mitochondrial arginine transporter BAC2-like”, participate in producing ornithine/agmatine from arginine [57,58].

**Figure 9:**
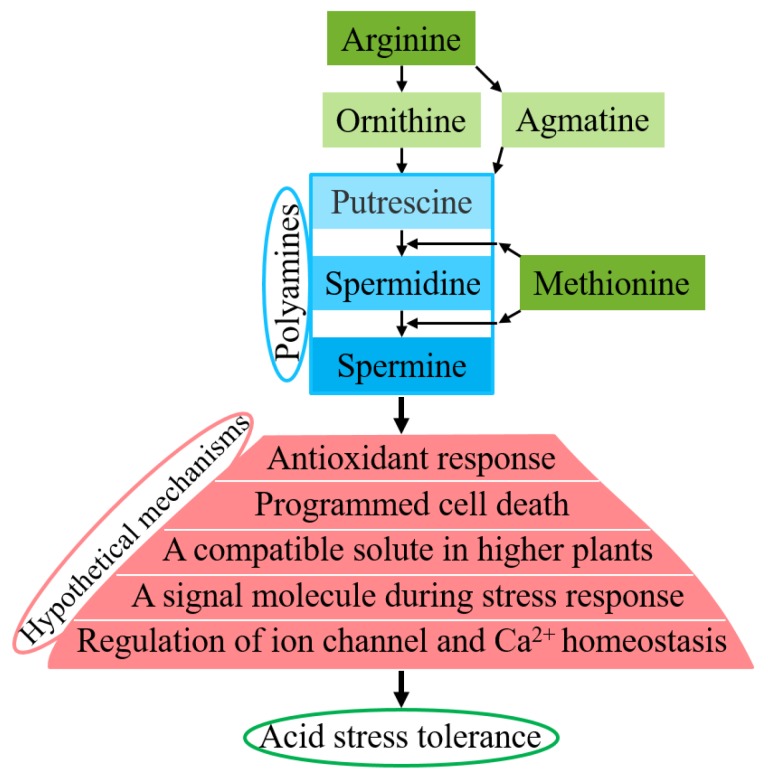
Overview of hypothetical regulatory mechanisms of enhanced acid stress tolerance via polyamines. Acid stress induces the accumulation of polyamines, which then function in antioxidant responses, in programmed cell death, as compatible solutes in higher plants, as signaling molecules during stress responses, in the regulation of ion channels and Ca^2+^ homeostasis, and in other unknown pathways, by scavenging excess free radicals or stabilizing membranes by binding to negatively charged groups, leading to enhanced acid stress tolerance.

The hypothetical regulatory mechanisms of enhanced acid stress tolerance via polyamines are illustrated in Figure 9. These varied hypothetical mechanisms were proposed by Liu et al. [53], Hussain et al. [9], Gupta et al. [11], Taie et al. [16], and Zarza et al. [17]. The biosynthesis of polyamines in plants has been well demonstrated [59,60,61,62,63,64,65]. Putrescine is produced either directly from ornithine or indirectly from arginine. Putrescine is converted into spermidine, and spermidine is converted into spermine, both of which require methionine. Polyamines include putrescine (diamine), spermidine (triamine), and spermine (tetramine). The hypothetical roles of polyamines include antioxidant responses, programmed cell death, acting as a compatible solute in higher plants, acting as a signaling molecule during stress responses, regulating ion channels and Ca^2+^ homeostasis, and other unknown pathways. Previous works [4,9,11,15,16,17,18,20,43,46,52,53,62] have indicated that accumulated polyamines may play vital roles in counteracting stress (1) as antioxidants, free radical scavengers, and membrane stabilizers that can interact freely with DNA/RNA/proteins/membrane lipids to prevent the membrane system from denaturing under acid stress conditions; (2) through the cationic nature of spermine^+4^ > spermidine^+3^ > putrescine^+2^ at physiological pH and/or improvement of the K^+^/Ca^2+^ ion balance; (3) as cellular signals in intrinsic cross-talk with abscisic acid (ABA), hydrogen peroxide, and nitrous oxide during acid stress responses by a complex network; and (4) by increasing cell proliferation, decreasing apoptosis and increasing the expression of genes associated with tumor invasion and metastasis, thus making their metabolism a target for cancer treatment and prevention.

## 5. Conclusions

In summary, this study provides a comprehensive analysis of the acid-responsive genes and transcriptome expression profiles of iron walnut leaves via a combination of RNA-seq, STEM profiling, the ML method, and physiological approaches. Our results revealed 1249 potentially acid-responsive genes in iron walnut, which we annotated by using the GO and KEGG databases, showing that polyamine accumulation enhanced iron walnut acid stress tolerance. These results fill the knowledge gap in the available literature concerning acid resistance-related genes in iron walnut. Next, we seek to understand how environmental changes (such as acid stress) are communicated via cellular signal transduction to induce a coordinated metabolic response and how the function of polyamines is influenced by transcriptional and post-translational modifications.

## Figures and Tables

**Table 1:** Summary of the RNA-seq data obtained from different samples.

Sample	Clean Reads	Q30	GC	Mapped Genome	Unique Mapped Genome	Mapped Genes	Expressed Genes
pH 3–4_1	28,020,622	94.93%	45.98%	87.60%	68.87%	75.69%	26,186
pH 3–4_2	26,738,560	96.36%	46.37%	82.25%	63.00%	69.82%	28,134
pH 3–4_3	27,011,236	96.32%	46.85%	85.60%	66.22%	73.12%	28,922
pH 4–5_1	27,605,126	96.02%	46.39%	85.75%	68.97%	74.31%	27,392
pH 4–5_2	27,634,854	96.44%	47.05%	88.56%	70.73%	75.93%	27,670
pH 4–5_3	27,109,750	96.50%	46.59%	87.53%	69.72%	75.85%	27,582
pH 5–6_1	26,864,464	96.47%	46.51%	87.59%	71.06%	73.90%	26,644
pH 5–6_2	27,448,384	96.36%	46.37%	84.86%	67.01%	73.6%	27,525
pH 5–6_3	27,338,826	96.40%	46.60%	87.13%	68.69%	75.66%	26,942
pH 6–7_1	27,698,734	94.98%	46.42%	88.08%	68.34%	76.53%	26,864
pH 6–7_2	27,894,576	96.50%	46.45%	84.43%	66.27%	72.39%	27,221
pH 6–7_3	27,288,782	96.36%	46.73%	86.17%	69.15%	72.39%	26,706

**Table 2:** Primers for qRT-PCR assays used for the twelve RNA-seq libraries in this study.

Gene ID	Forward Primer (5′ to 3′)	Reverse Primer (5′ to 3′)	Amplicon Size (bp)
gene17916	GAAGGCGAAGAAGAAGAAGA	CTGGCGGTAACTGTAACTC	96
gene22045	GGCGTGAAGGAGTTGATT	ACAGTGTTAAGGTCGTATCG	97
gene25150	AAGACGATGTTGATGATTCC	TTCCAGTATTAGCGGTAAGA	100
gene25184	TGAGGCTGAAGAGTATGC	CGTAGATGGTTGGATGGT	82
gene29464	CTGTGTTGTGGTAGAGGA	TCTTCATCGGCTGTGTAA	81
gene29846	GAGAAGGCTATCACAAGAAG	CCAGTATGACAAGGAGTAATC	113
gene31088	GATGCTGTGTTGCTGTTC	CCGCCATTATCTGCTTGA	131
gene31799	GCTATAACTACGCTATATTCG	TTACTTCTGATTCTCCTATGT	111
gene35192	GCTGGAAGTCATAGTAAGG	ATGGCTGCTAATCACAAG	167
gene39645	GAGTGGGAATGAAGGAAGA	ATTGGCAGAGGAATTGGA	72

**Table 3:** Pathways from six cross-comparisons between the different gene sets.

Pathway ID	Pathway	FDR < 0.05	No. DEGs
ko01200	Carbon metabolism	5.70 × 10^−7^	50
ko01100	Metabolic pathways	5.70 × 10^−7^	229
ko01110	Biosynthesis of secondary metabolites	1.65 × 10^−6^	146
ko00710	Carbon fixation in photosynthetic organisms	1.83 × 10^−6^	28
ko00630	Glyoxylate and dicarboxylate metabolism	8.98 × 10^−6^	17
ko00908	Zeatin biosynthesis	2.25 × 10^−5^	14
ko00195	Photosynthesis	1.20 × 10^−4^	12
ko00010	Glycolysis/gluconeogenesis	1.04 × 10^−3^	29
ko03430	Mismatch repair	1.81 × 10^−3^	2
ko03050	Proteasome	1.81 × 10^−3^	1
ko00220	Arginine biosynthesis	2.13 × 10^−3^	10
ko00903	Limonene and pinene degradation	4.64 × 10^−3^	14
ko00910	Nitrogen metabolism	4.64 × 10^−3^	10
ko00030	Pentose phosphate pathway	4.64 × 10^−3^	20
ko01230	Biosynthesis of amino acids	5.00 × 10^−3^	35
ko00071	Fatty acid degradation	5.88 × 10^−3^	6
ko00511	Other glycan degradation	6.71 × 10^−3^	1
ko00500	Starch and sucrose metabolism	7.97 × 10^−3^	40
ko00670	One carbon pool by folate	8.93 × 10^−3^	7
ko04075	Plant hormone signal transduction	1.07 × 10^−2^	43
ko00260	Glycine, serine and threonine metabolism	1.22 × 10^−2^	13
ko00250	Alanine, aspartate and glutamate metabolism	1.42 × 10^−2^	9
Ko00330	Arginine and proline metabolism	1.42 × 10^−2^	7
ko00051	Fructose and mannose metabolism	1.42 × 10^−2^	17
ko03015	mRNA surveillance pathway	1.60 × 10^−2^	1
ko03040	Spliceosome	1.60 × 10^−2^	2
ko01040	Biosynthesis of unsaturated fatty acids	2.06 × 10^−2^	1
ko00945	Stilbenoid, diarylheptanoid and gingerol biosynthesis	2.06 × 10^−2^	4
ko00450	Selenocompound metabolism	2.15 × 10^−2^	7
ko00740	Riboflavin metabolism	2.25 × 10^−2^	4
ko04626	Plant-pathogen interaction	2.72 × 10^−2^	51
ko00052	Galactose metabolism	2.90 × 10^−2^	12
ko00943	Isoflavonoid biosynthesis	2.90 × 10^−2^	1
ko00240	Pyrimidine metabolism	2.90 × 10^−2^	2
ko00592	α-linolenic acid metabolism	3.33 × 10^−2^	6
ko00196	Photosynthesis-antenna proteins	4.07 × 10^−2^	4
ko00942	Anthocyanin biosynthesis	4.44 × 10^−2^	6
ko04141	Protein processing in endoplasmic reticulum	4.44 × 10^−2^	40

**Table 4:** Six genes closely related to polyamine biosynthesis/metabolism.

Gene ID	Descriptions	
	GO Terms	KEGG Pathways
gene42780	spermidine hydroxycinnamoyl transferase-like	
	----	ko01110, Biosynthesis of secondary metabolites
		ko01100, Metabolic pathways
		ko00945, Stilbenoid, diarylheptanoid and gingerol biosynthesis
		ko00941, Flavonoid biosynthesis
		ko00940, Phenylpropanoid biosynthesis
gene28192	spermine synthase-like	
	GO:0003674, molecular function GO:0003824, catalytic activity	ko00410, β-Alanine metabolism
		ko00480, Glutathione metabolism
		ko00270, Cysteine and methionine metabolism
		ko00330, Arginine and proline metabolism
		ko01100, Metabolic pathways
gene4089	S-adenosylmethionine synthase 1-like	
	----	ko01230, Biosynthesis of amino acids
gene31260	arginine decarboxylase-like	
	----	ko00330, Arginine and proline metabolism
gene24987	serine/arginine-rich splicing factor RS40-like	
	----	ko03040, Spliceosome
gene9388	mitochondrial arginine transporter BAC2-like	
	GO:0016053, organic acid biosynthetic process	ko04714, Thermogenesis
	GO:0046394, carboxylic acid biosynthetic process	
	GO:0005488, binding	
	GO:0006810, transport	

## Supplementary Materials

The following are available online at https://www.mdpi.com/2073-4425/10/8/605/s1, Table S1: Gene expression for each sample. The first column in the table presents the gene ID, and the first row in the table presents the treatment and gene descriptions. (XLSX 3.32 MB); Table S2 Short Time-series Expression Miner (STEM) cluster analysis. The first column in the table presents the gene ID, and the first row in the table presents the treatment and gene descriptions (XLSX 311 kB).
